# Assessment of eating disorders with the diabetes eating problems survey – revised (DEPS-R) in a representative sample of insulin-treated diabetic patients: a validation study in Italy

**DOI:** 10.1186/s12888-017-1434-8

**Published:** 2017-07-19

**Authors:** Federica Pinna, Enrica Diana, Lucia Sanna, Valeria Deiana, Mirko Manchia, Eraldo Nicotra, Andrea Fiorillo, Umberto Albert, Alessandra Nivoli, Umberto Volpe, Anna Rita Atti, Silvia Ferrari, Federica Medda, Maria Gloria Atzeni, Daniela Manca, Elisa Mascia, Fernando Farci, Mariangela Ghiani, Rossella Cau, Marta Tuveri, Efisio Cossu, Elena Loy, Alessandra Mereu, Stefano Mariotti, Bernardo Carpiniello

**Affiliations:** 1 Department of Public Health, Clinical and Molecular Medicine, University of Cagliari, Cagliari, Italy; 2 Department of Pharmacology, Dalhousie University, Halifax, NS Canada; 3 Department of Pedagogy, Psychology, Philosophy, University of Cagliari, Cagliari, Italy; 4 Department of Psychiatry, University of Naples SUN, Naples, Italy; 5 Rita Levi Montalcini Department of Neuroscience, Anxiety and Mood Disorders Unit, University of Turin, Turin, Italy; 6 Psychiatric Institute, Department of Clinical and Experimental Medicine, University of Sassari, Sassari, Italy; 7 Department of Biomedical and Neuro Motor Sciences, University of Bologna, Bologna, Italy; 8 Department of Diagnostic-Clinical Medicine and Public Health, University of Modena & Reggio Emilia, Modena, Italy; 9 Unit of Diabetology, ASL-Cagliari, Quartu Sant’Elena, Italy; 10 Endocrinology and Diabetes Unit, AOU-Cagliari, Monserrato, Italy; 11 Department of Medical Sciences, University of Cagliari, Monserrato, Italy

**Keywords:** Eating disorder, Diabetes, Disturbed eating behavior, Insulin manipulation

## Abstract

**Background:**

The purpose of the study was to evaluate in a sample of insulin-treated diabetic patients, with type 1 or type 2 diabetes, the psychometric characteristics of the Italian version of the DEPS-R scale, a diabetes-specific self-report questionnaire used to analyze disordered eating behaviors.

**Methods:**

The study was performed on 211 consecutive insulin-treated diabetic patients attending two specialist centers. Lifetime prevalence of eating disorders (EDs) according to DSM-IV and DSM-5 criteria were assessed by means of the Module H of the Structured Clinical Interview for DSM IV Axis I Disorder and the Module H modified, according to DSM-5 criteria. The following questionnaires were administered: DEPS-R and the Eating Disorder Inventory – 3 (EDI-3). Test/retest reproducibility was assessed on a subgroup of 70 patients. The factorial structure, internal consistency, test-retest reliability and concurrent validity of DEPS-R were assessed.

**Results:**

Overall, 21.8% of the sample met criteria for at least one DSM-5 diagnosis of ED. A “clinical risk” of ED was observed in 13.3% of the sample. Females displayed higher scores at DEPS-R, a higher percentage of at least one diagnosis of ED and a higher clinical risk for ED. A high level of reproducibility and homogeneity of the scale were revealed. A significant correlation was detected between DEPS-R and the 3 ED risk scales of EDI-3.

**Conclusions:**

The data confirmed the overall reliability and validity of the scale. In view of the significance and implications of EDs in diabetic patients, it should be conducted a more extensive investigation of the phenomenon by means of evaluation instruments of demonstrated validity and reliability.

**Electronic supplementary material:**

The online version of this article (doi:10.1186/s12888-017-1434-8) contains supplementary material, which is available to authorized users.

## Background

The management of diabetes patients needs to pay careful attention to the food they consume, to refer to nutritional tables, to apply precision in the preparation of portions and to constantly monitor calorie intake on the basis of insulin dosage. However, this focus on food, fundamental in controlling the disease, places diabetic patients at increased risk of developing EDs. However, EDs appear to be harder to identify in this category of patients, and distinguishing between a healthy eating regime and a disturbed behavior may prove to be an arduous task [1]. Accordingly, considerable interest has been shown in the in-depth analysis of the significance and characteristics of clinical entities widely referred to as “Disturbed Eating” or “Disturbed Eating Behaviors” (DEB), which are generally considered borderline categories of ED made up of symptoms that have not yet reached a level of severity and frequency to allow them to be classified as EDs [2]. Frequently a DEB will be manifested only as a change in the eating pattern of diabetic patients [3]. Reports present in the literature are largely in agreement over the high rates of EDs, DEB, binge eating and bulimic symptoms observed in patients with both type 1 [4–13], and type 2 diabetes [8, 14], reporting generally higher rates of EDs in diabetic patients compared to the general population [1, 4, 6, 7, 13, 15]. A recent review and meta-analysis of 6 ED studies and 8 focusing on DEB, reported a higher prevalence for both conditions amongst adolescents and young adults with type 1 diabetes than in age-matched non-diabetic controls [13]. Jones et al. [7] highlighted the presence of EDs in 10% of adolescents with type 1 diabetes aged between 12 and 19 years, versus 4% of healthy age-matched individuals. Neumark-Sztainer et al. [10] found high rates of DEB (37.9% in females and 15.9% in males) in adolescents and young adults with type 1 diabetes aged from 12 to 21 years. These EDs tend to first present during adolescence and persist into adulthood, particularly if left untreated [5]. The fact that the vast majority of studies are focused on young females, and that studies regarding adults with diabetes are somewhat scarce, makes it highly likely that a large number of cases of EDs in this population may be underestimated and under-diagnosed [16]. Within the range of specific DEBs in insulin-treated diabetic patients, insulin manipulation is of particular interest. This practice, defined by several authors as “diabulimia”, may be viewed as the analog of an ED in which adolescents and young adults, mostly females, with insulin-treated diabetes deliberately omit to administer insulin, manipulating the prescribed dose with the aim of losing weight or preventing weight gain [17]. Those in charge of diabetes management have long acknowledged this practice, and prevalence rates are so high to suggest the need to recognize diabulimia as an overt psychiatric disorder with specific diagnostic criteria, suitable for inclusion as a stand-alone clinical entity in the classifications of psychiatric diagnostic manuals [18]. The restriction or omission of insulin to control body weight is considered a form of purging available solely to diabetic patients, compromises control of the metabolism and determines an increased risk of morbidity and mortality [19]. Some of the more commonly observed alarm signals, which may point to the presence of insulin manipulation, include: persistently high glycosylated hemoglobin levels, frequent referral to the emergency department for diabetic ketoacidosis, tendency towards thinness, dissatisfaction with personal body image, hyperglycemia with frequent candidiasis or urinary infections, and poor compliance with check-up visits and treatments for diabetes [17]. The first article reporting on insulin purging in a diabetic patient was published in 1983 [20]. Subsequently, an increasing number of studies have investigated the presence of insulin omission in samples of diabetic patients, attempting to estimate the prevalence and diffusion of this specific behavior. The findings demonstrate how prevalence increases with age, rising from 1% in pre-adolescence [4], to 11–14% during the initial stages of adolescence [7, 21], reaching 27–39% in late adolescence and early adulthood [5, 21, 22]. A study conducted by Ackard et al. on 143 adolescents, 73 males and 70 females, affected by type 1 diabetes, found that 10.3% of females omitted to administer insulin, and 7.4% administered a lower dose, in order to control their weight, versus 1.4% of males [23]. Another significant aspect currently subjected to increasing debate relates to the efficacy of traditional screening tools for ED in this patient population. Powers et al. reported how both the Eating Disorders Examination Questionnaire (EDE-Q) and the Eating Disorders Inventory, version 3 (EDI – 3), when administered to subjects with diabetes, may provide scarcely reliable results [24]. Indeed, due to the emphasis placed on the careful monitoring of food intake and weight control during diabetes treatment, several items may be misinterpreted by patients resulting in a consequent increased risk of both false positives and false negatives. To date, the only two specific questionnaires for use in investigating ED in the diabetic population are the Diagnostic Survey for Eating Disorders (DSED) and the Diabetes Eating Problems Survey [DEPS], both of which include items specifically correlated to diabetes, such as insulin manipulation for the purpose of weight control. The DSED is a self-evaluation tool comprising 12 sections, although its use as a structured interview has also been suggested [25]. A modified version of this scale, the DSED – M, has subsequently been developed [22, 26]. The DEPS is a questionnaire comprising 28 items developed in 2001 by Antisdel et al. [27]. Initially tested on adult female patients with type 1 diabetes over the age of 18 years, the scale has subsequently been used as an evaluation tool in populations of adolescent diabetic patients [28]. In 2010 Markowitz et al. validated a revised, shortened version of DEPS, DEPS-R, comprising 16 items, in a sample of 112 adolescents with type 1 diabetes, 49 males and 63 females, in an age range of 13–19 years [29]. The DEPS-R scale, developed as a tool capable of rapidly screening for EDs in a pediatric population with type 1 diabetes, is currently recognized as a valid screening tool for use in identifying at-risk EDs in this patient population [1, 16, 29–32]. In view of the relevance of EDs in diabetic subjects and the associated health risks, the need for further in-depth studies to be conducted on an extended clinical sample of diabetic patients, both children and adults, with both type 1 and type 2 diabetes using specific questionnaires of demonstrated validity and reliability, should be underlined. Considering these premises, the purpose of the present study was to validate the Italian version of the DEPS-R scale in a sample of insulin-treated male and female subjects with type 1 and type 2 diabetes aged from 13 to 55 years. The authors aimed to overcome some of the more significant limitations of previous validation studies, extending validation to adults and to subjects affected by type 2 diabetes, and analyzing the DEPS-R scale in relation to a self-administered specific tool for the assessment of EDs accompanied by the use of a structured clinical interview for the diagnosis of EDs according to DSM-IV and DSM 5 criteria [29, 31, 32].

Taking into account the high prevalence and implications of comorbidity between diabetes and EDs, and the singular means of manifestation of EDs in diabetic subjects, the availability of specific screening tools of proven validity is crucial in facilitating the development of timely and effective multidisciplinary interventions aimed at minimizing the short and long-term risk of complications.

## Methods

A cross-sectional study was performed in a representative sample of insulin-treated diabetic patients with type 1 or type 2 diabetes aged from 13 to 55 years. An unselected sample of consecutive insulin-treated diabetic patients attending two specialist centers for the diagnosis and treatment of diabetes over a 4-month period was assessed. These centers usually provide a global medical and psychological evaluation based on a multidisciplinary approach. When necessary, psychological support or psychiatric interventions are provided. The data used for this study derived from the routine assessment of psychopathology, by means of a structured clinical interview and specific questionnaires used to assess EDs, in the diabetic patients attending the specialist centers. Residents in psychiatry conducted the interviews, after having been trained in the use of the instrument by a senior psychiatrist (FP). Inter-rater reliability, evaluated using Cohen’s K before starting the study, remained substantial (K > 0.80). The study group was made up of 211 participants (13–55 years old; 108 males, 103 females), who met the above inclusion criteria.

Socio demographic data, medical history and clinical data were collected by means of a structured interview. Lifetime prevalence of EDs according to DSM-IV and DSM-5 criteria were assessed by means of the Module H of the Structured Clinical Interview for DSM IV Axis I Disorder (SCID-I, Research Version, Non-Patient Edition) [33] and the Module H modified, according to DSM-5 criteria. The rationale for performing diagnosis using either DSM IV or DSM-5 criteria was related to the acknowledged increased ability of DSM-5 criteria to identify the presence of an ED and to formulate a “specified ED diagnosis”, thus reducing the rate of “non – specified ED diagnosis” [34].

The following questionnaires were administered: DEPS-R [29] and the EDI-3 [35]. Test/retest reproducibility of DEPS-R was evaluated on a subgroup of 70 patients who filled the questionnaire in twice at approximately one-month intervals.

### Instruments

#### DEPS-R

DEPS-R is a diabetes-specific measure of DEB. The instrument is self-administered and composed of 16 items on a 6-point Likert, ranging from 0 to 5, in relation to frequency of the behavior (0 = never; 1 = rarely; 2 = sometimes; 3 = often; 4 = usually; 5 = always). It can be completed in less than 10 min. Higher scores indicate more DEB. The original instrument comprised 28 items but it was lately revised and shortened to the DEPS-R by Markowitz et al. [29]. The original DEPS-R was found to have good psychometric properties in two different samples of young people (11–19 years) with type 1 diabetes [29, 32]. More recently, the German version of the scale was also found to have good psychometric properties in a sample of young people, aged 11–19 years, with type 1 diabetes [31]. Relating to the aim of this study, a bilingual psychiatrist, with expertise in the area of ED, translated the original English version of the scale into Italian. A bilingual native English translator, blind to the original version of the tool, later back translated the scale into English. A high degree of similarity between the English translation and the original version was found. (See Additional file 1 for a copy of the Italian version of the questionnaire).

#### EDI-3

EDI-3 is a self-administered scale created in order to identify subjects at risk of developing an ED. The questionnaire analyzes psychological traits and key symptoms considered relevant in the development and maintenance of EDs. It is composed of 91 items with a choice of 6 answers, based on frequency of the behavior (A = always; B = usually; C = often; D = sometimes; E = rarely; F = never), 12 primary scales (3 ED risk scales, 9 psychological scales) and six composite scores. High scores at ED risk scales (“Drive for Thinness”, DT; “Bulimia”, B; “Body Dissatisfaction”, BD), are linked to an increased risk of developing an ED. At each scale, a percentile score ranging from 70° to 85° and a percentile score exceeding 85° identify, respectively, the presence of a clinical and a high clinical risk in the area evaluated. The Eating Disorder Risk Composite (EDRC) score derives from a combination of the three ED risk scales and provides a global measure of ED risk and of concerns with food and body weight.

#### Module H of the SCID-I

Lifetime prevalence of ED according to DSM-IV and DSM-5 criteria were assessed by means of the Module H of the Structured Clinical Interview for DSM IV Axis I Disorder (SCID-I, Research Version, Non-Patient Edition) [33] and the Module H modified, according to DSM-5 criteria. It was possible to investigate the following diagnostic categories: Anorexia Nervosa and Bulimia Nervosa according to DSM-IV and DSM-5 criteria, Binge Eating Disorder according to DSM-5 criteria, Eating Disorder Not Otherwise Specified according to DSM-IV criteria, Unspecified Eating Disorder and Other Specified Eating Disorder according to DSM-5 criteria (the modified version of Module H is available from authors).

### Statistical analyses

To retest construct validity of the Italian version of DEPS-R, a Confirmatory Factor Analysis was performed [36]. A value of commonality was calculated for each item. Intraclass correlation coefficient (ICC) was used to estimate test-retest reliability. Internal consistency of the DEPS-R and EDI-3 scales were evaluated by Cronbach’s Alpha coefficient. Concurrent validity was studied by correlating the DEPS-R scale with EDI-3 by means of Pearson’s correlation coefficient. The incremental validity of DEPS-R and EDI-3 scales (predictor variables) versus the dichotomous variable DSM-5 (criterion variable) was assessed by means of hierarchical logistic regression [37, 38] using statistical package R. A correlation between scores obtained at DEPS-R and at EDI-3 for EDCR and relevant clinical variables was carried out using Pearson’s coefficient of linear correlation for continuous scales. Continuous and categorical variables were compared using, as appropriate, Mann-Whitney test, Pearson’s chi-squared test and z-test. Statistical significance was set at *p* < 0.005. Data analysis was carried out using SPSS software - version 20.0, LISREL version 8.80 and R Software version 3.3.0.

## Results

### Characteristics of the study population

The sample studied included 211 insulin-treated diabetic patients, 192 patients with a type 1, 19 patients with a type 2 diabetes, 108 males and 103 females, with a median age of 38 years (13–55 years old), a median Body Max Index (BMI) of 24 and a median score at DEPS-R of 12 (Table 1). Overall, 21.8% of the sample met criteria for at least one DSM-5 diagnosis of ED, 12.8% of the sample met criteria for at least one DSM-IV diagnosis of ED (Table 1). Considering the percentiles of EDRC composite score, a “clinical risk” of ED was observed in 13.3% of the sample.

**Table 1:** Descriptive data

	TOTAL	MALE	FEMALE	*p*	*U* di Mann-Whitney	*z*-value	I.C. 95%	
SAMPLE	211	108	103					
MEDIAN AGE (median)	38	38	38	0.74	5417.5			
YEARS OF STUDY (median)	13	13	13	0.33	5142.0			
BMI (median)	24	24	23	0.11	485.5			
DEPS TOTAL(median)	12	10	14	0.004	4283.5			
DSM-IV (perventage)	27 (12.8%)	9 (8.3%)	18 (17.5%)	0.0467		1.991	0.014	0.181
DSM-5 (perventage)	46 (21.8%)	12 (11.1%)	34 (33.0%)	0.00008		3.958	0.111	0.327
DIABETES TYPE I (perventage)	192 (91.0%)	97 (89.8%)	95 (92.2%)	0.54				
DIABETES TYPE II (perventage)	19 (9.0%)	11 (10.2%)	8 (7.8%)			0.616	−0.053	0.101

Data are medians and percentages, *p* values refer to the significance of the difference between male and female (Mann-Whitney *U*-test and z-test respectively)

### DEPS-R and EDI-3 scores and EDs according to DSM-IV and DSM-5 criteria relating to gender

Females showed significantly higher scores at DEPS-R (median = 14) in relation to males (median = 10) (Table 1). Similarly, a significant higher percentage of females met criteria for at least one DSM-5 or DSM-IV diagnosis of ED (33% and 17.5% respectively), than that observed in men (11.1% and 8.3% respectively) (Table 1). Based on the percentiles of EDRC composite score and of “Drive for Thinness” and “Body Dissatisfaction” scales, a “clinical risk” for ED was detected in a higher percentage of females (22.3%, 21,4% and 28.2% respectively) compared to males (4.67%, 4.67% and 7.48% respectively) (CI = 0.087–0.27, *p* = 0.0001; CI = 0.078, *p* = 0.0001; CI = 0.106–0.307, *p* = 0.0001 respectively). No statistically significant gender difference was observed in prevalence of “clinical risk” at “Bulimia”.

### DEPS-R score relating to ED diagnosis according to DSM-IV and DSM-5 criteria

Subjects who met criteria for at least one DSM-5 or DSM-IV diagnosis of ED obtained significantly higher scores at DEPS-R (median = 22 and 21 respectively), compared to subjects without an ED diagnosis (median = 10) (*p* < 0.0001).

### Confirmatory factor analysis

**Fig. 1:**
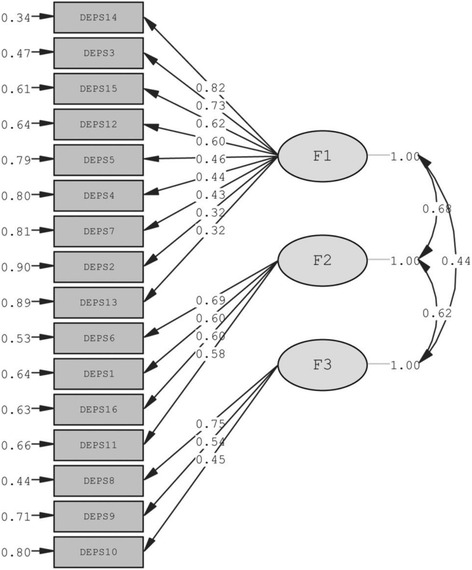
Path-diagram of the factorial solution

To retest construct validity of the Italian version of DEPS-R, a Confirmatory Factor analysis was performed (Tables 2, 3; Fig. 1). The latent structure of subscales was found to fully conform to that reported by Wisting et al. [32] during validation of the original scale. Indeed, the 3 latent factors relating to dimensions “maladaptive eating habits” (factor 1), “preoccupation with thinness or weight” (factor 2), and “concept of maintaining high blood glucose values to lose weight” (factor 3) were all confirmed (Table 2) [32]. All λ^x^ structural parameters were significantly different from zero in the respective t-tests performed (Table 2). Figure 1 illustrates the path-diagram of the factorial solution obtained. Similar to previous findings [32], the latent dimensions of DEPS-R were found to be significantly inter-correlated, although these correlations failed to reach high values under any condition (Fig. 1). Table 3 reports the main goodness of fit statistics of the model. The results obtained by the analysis demonstrate that the confirmatory factor analysis cannot be rejected in the light of the estimated goodness of fit statistics of the model. Indeed, the relative chi-square statistic, corrected for the degree of freedom of the model, yields a score in line with the acceptability criteria of the factorial solution (χ^2^/df = 250.83/101 = 2.48) [39, 40]. Likewise, high scores are also obtained by the other goodness of fit statistics, thus confirming the satisfactory statistical validity of the factorial model (GFI = 0.87; AGFI = 0.82; RMSEA = 0.085; FI = 0.85; NNFI = 0.88) (Table 3). In this context, the high values yielded by coefficients of reproducibility of the factorial solution should also be highlighted (CFI = 0.90; IFI = 0.90), thus demonstrating the substantial stability of the structural parameters of the model even in samples characterized by different social and anagraphic characteristics, although structurally homogenous to the sample on which DEPS-R was originally validated under the constraint of configural invariance of the factorial solution [40, 41].

**Table 2:** Confirmatory Factor structure of the Diabetes Eating Problem Survey Revised - DEPS-R

	F1	F2	F3
D 14 (I feel that my eating is out of control)	*λ* ^*x*^_*11*_ *= 0.82*		
D 3 (Other people have told me that my eating is out of control)	*λ* ^*x*^_*21*_ *= 0.72*		
D 15 (I alternate between eating very little and eating huge amounts)	*λ* ^*x*^_*31*_ *= 0.62*		
D12 (Other people tell me to take better care of my diabetes)	*λ* ^*x*^_*41*_ *= 0.59*		
D 5 (I eat more when I am alone than when I am with others)	*λ* ^*x*^_*51*_ *= 0.45*		
D 4 (When I overeat, I don’t take enough insulin to cover the food)	*λ* ^*x*^_*61*_ *= 0.44*		
D 7 (I avoid checking my blood sugar when I feel like it is out of range)	*λ* ^*x*^_*71*_ *= 0.43*		
D 2 (I skip meals and/or snacks)	*λ* ^*x*^_*81*_ *= 0.32*		
D 13 (After I overeat, I skip my next insulin dose)	*λ* ^*x*^_*91*_ *= 0.32*		
D 6 (I feel that it’s difficult to lose weight and control my diabetes at the same time)		*λ* ^*x*^_*12*_ *= 0.68*	
D 16 (I would rather be thin than to have good control of my diabetes)		*λ* ^*x*^_*22*_ *= 0.61*	
D 1 (Losing weight is an important goal to me)		*λ* ^*x*^_*32*_ *= 0.60*	
D11 (I feel fat when I take all of my insulin)		*λ* ^*x*^_*42*_ *= 0.59*	
D 8 (I make myself vomit)			*λ* ^*x*^_*13*_ *=* *0.75*
D 9 (I try to keep my blood sugar high so that I will lose weight)			*λ* ^*x*^_*23*_ *=* *0.54*
D 10 (I try to eat to the point of spilling ketones in my urine)			λ^x^_33_ = 0.44

Data represent pattern coefficients

**Table 3:** CFA Goodness of fit statistics

Degrees of Freedom = 101	
Relative Chi-Square = 250.8.83/101 = 2.483	
Root Mean Square Error of Approximation (RMSEA) = 0.085	
90 Percent Confidence Interval for RMSEA = (0.072; 0.098)	
Normed Fit Index (NFI) = 0.85	
Non-Normed Fit Index (NNFI) = 0.88	
Comparative Fit Index (CFI) = 0.90	
Incremental Fit Index (IFI) = 0.90	
Root Mean Square Residual (RMR) = 0.073	
Goodness of Fit Index (GFI) = 0.87	
Adjusted Goodness of Fit Index (AGFI) = 0.82

### Item-total correlations

The communality values (item-total correlations), means and standard deviations for each item of DEPS-R are listed in Table 4.

**Table 4:** The communality value, the average and the standard deviation of each item of DEPS-R item statistics for total scale

	Item-total r	Mean	Standard deviation
D 1 (Losing weight is an important goal to me)	0,68	2.21	1.809
D 2 (I skip meals and/or snacks)	0.26	1.22	1.256
D 3 (Other people have told me that my eating is out of control)	0.63	1.09	1.361
D 4 (When I overeat. I don’t take enough insulin to cover the food)	0.45	1.01	1.168
D 5 (I eat more when I am alone than when I am with others)	0.54	0.88	1.286
D 6 (I feel that it’s difficult to lose weight and control my diabetes at the same time)	0.6	1.55	1.652
D 7 (I avoid checking my blood sugar when I feel like it is out of range)	0.58	0.71	1.17
D 8 (I make myself vomit)	0.58	0.04	0.246
D 9 (I try to keep my blood sugar high so that I will lose weight)	0.44	0.12	0.494
D 10 (I try to eat to the point of spilling ketones in my urine)	0.62	0.03	0.205
D 11 (I feel fat when I take all of my insulin)	0.59	0.33	0.945
D 12 (Other people tell me to take better care of my diabetes)	0.54	1.85	1.607
D 13 (After I overeat. I skip my next insulin dose)	0.58	0.12	0.416
D 14 (I feel that my eating is out of control)	0.67	1.04	1.3
D 15 (I alternate between eating very little and eating huge amounts)	0.53	0.9	1.116
D 16 (I would rather be thin than to have good control of my diabetes)	0.56	0.46	1.105

### Test-retest reliability

Test-retest reliability revealed a high degree of reproducibility for DEPS-R: ICC = 0.950 (I.C 95%: 0.919–0.969).

### Internal consistency

Analysis of internal consistency of responses to each single item of DEPS-R pointed out a good homogeneity for the scale (Cronbach’s Alpha = 0.83). Cronbach’s Alpha for the EDRC composite score at EDI-3 was 0.95.

### Concurrent validity

Correlations between DEPS-R and EDI-3 are shown in Table 5. A significant correlation was observed between total scores obtained at DEPS-R, the 3 ED scales and the EDRC at EDI-3. Factor 1, Factor 2 and Factor 3 correlated significantly with both ED scales and EDRC at EDI-3.

**Table 5:** Correlation among scales

		DEPS-R total	Factor 1	Factor 2	Factor 3	Drive for thinness	Bulimia	Body dissatisfaction	EDRC
1	DEPS-R total								
2	Factor 1	0.925**							
3	Factor 2	0.859**	0.671**						
4	Factor 3	0.596**	0.437**	0.624**					
7	Drive for thinness	0.631**	0.556**	0.698**	0.399**				
8	Bulimia	0.556**	0.591**	0.423**	0.333**	0.578**			
9	Body dissatisfaction	0.525**	0.462**	0.613**	0.206**	0.608**	0.392**		
10	EDRC	0.673**	0.618**	0.715**	0.351**	0.866**	0.688**	0.89**	

**p < 0.0001

### Correlation of DEPS-R and EDRC with relevant clinical variables

The DEPS-R total value positively correlated with BMI median score in the sample as a whole and in males, females, subjects with type 1 and subjects with type 2 diabetes (Table 6). The DEPS-R total value positively correlated with HbA1C value in the sample as a whole and in males, females and subjects with type 1 diabetes (Table 6). No significant correlation with age, age of onset of diabetes, diabetes duration and number of injections of insulin per day was found. The EDRC score positively correlated with BMI median score in the sample as a whole and in males, females and subjects with type 1 diabetes (Table 6). No significant correlation with age, HbA1C value, age of onset of diabetes, diabetes duration and number of injections of insulin per day was found (Table 6).

**Table 6:** Correlation of DEPS-R with relevant variables

	DEPS-R					EDCR				
	r (*p*)					r (*p*)				
	Whole cohort	Males	Females	Subject with type 1 diabetes	Subject with type 2 diabetes	Whole cohort	Males	Females	Subject with type 1 diabetes	Subject with type 2 diabetes
Age	−0.037 (0.593)	−0.037 (0.593)	−0.102 (0.304)	−0.076 (0.295)	−0.422 (0.072)	0.018 (0.798)	0.047 (0.632)	−0.175 (0.077)	−0.143 (0.049)	−0.443 (0.065)
BMI	0.408 (0.000)	0.408 (0.000)	0.457 (0.000)	0.345 (0.000)	0.593 (0.007)	0.405 (0.000)	0.412 (0.000)	0.464 (0.000)	0.391 (0.000)	0.326 (0.187)
HbA1C	0.225 (0.001)	0.225 (0.001)	0.243 (0.014)	0.216 (0.003)	0.238 (0.341)	0.91 (0.193)	0.168 (0.088)	0.097 (0.332)	0.098 (0.178)	−0.08 (0.76)
Diabetes Age of onset	−0.012 (0.863)	−0.012 (0.863)	0.02 (0.843)	−0.054 (0.453)	−0.239 (0.325)	−0.044 (0.527)	−0.017 (0.865)	−0.042 (0.671)	−0.117 (0.107)	−0.223 (0.373)
Diabetes duration	−0.023 (0.738)	−0.023 (0.738)	−0.122 (0.22)	−0.014 (0.842)	0.075 (0.762)	0.068 (0.325)	0.074 (0.449)	−0.124 (0.211)	−0.012 (0.865)	0.043 (0.866)
Number of injections of insulin per day	−0.09 (0.242)	−0.077 (0.487)	−0.122 (0.264)	0.084 (0.307)	−0.364 (0.126)	−0.042 (0.586)	−0.003 (0.976)	−0.104 (0.343)	0.089 (0.281)	−0.279 (0.263)

In bracketes the level of significativity

### Incremental validity

To test incremental validity of DEPS-R versus EDI-3, a general rating scale for EDs, represented by the EDRC composite score, simple and multiple logistic regression analysis was applied [37, 38], using diagnosis of ED according to DSM-5 as dichotomous dependent variable, and continuous variables represented by EDRC scores achieved at DEPS-R, as predictors. The results obtained, similar to observations made for the EDRC composite score, demonstrated a high validity of DEPS-R scale in predicting ED diagnosis, particularly in view of the high statistical significance of comparability of multiple logistic regression parameters (Table 7). A parameter of β_1_ = 0.0439 was estimated for the EDRC variable (SE = 0.01450, *p* < 0.01), and a parameter of β_2_ = 0.08105 (SE = 0.02712, *p* < 0.01) for the DEPS-R variable. Moreover, a combined use of the two scales was found to produce a synergic effect in predicting a diagnosis of ED, further reducing the risk of detection of false negatives (Table 7).

**Table 7:** Multiple Logistic Regression of EDRC and DEPS-R vs Diagnosis of ED according to DSM-5 criteria

	Estimate	Std.Error	zvalue	Pr(>|z|)
(Intercept)	−3.74132	0.47591	−7.861	3.8e-15***
EDRC	0.04397	0.01450	3.032	0.00243**
DEPS-R	0.08105	0.02712	2.989	0.00280**

***p < 0.0001

**p < 0.001

## Discussion

The main purpose of the present study was to validate the Italian version of the DEPS-R scale, extending validation to a representative sample of insulin-treated male and female subjects with type 1 and type 2 diabetes aged from 13 to 55 years. The DEPS-R scale was first developed as a tool capable of rapidly screening for ED in a pediatric population with type 1 diabetes, and is currently recognized as a good screening instrument in recognizing individuals at-risk for developing ED in this patient population [1, 16, 29–32]. Considering the limitations of previous studies, largely performed on adolescents with type 1 diabetes, we decided to extend investigations and validation of the questionnaire, in terms of both type of diabetes and age, to a more representative sample of insulin-treated diabetic subjects including individuals with type 2 diabetes and adults. Indeed, although literature data relating to ED and DEB in diabetic patients focus largely on subjects with type 1 diabetes [4–13], ED would seem to impinge heavily also on subjects with type 2 diabetes [8, 14]. At the same time, although these disorders are manifested largely during adolescence, they frequently persist, particularly if left untreated, into adulthood [5]. The lack of studies conducted on a population of adult diabetic subjects moreover increases the risk of an underestimation and under-diagnosis of the majority of cases of ED in this population [16]. Taking into account the high prevalence and implications of comorbidity between diabetes and EDs, the singular means of manifestation of EDs in diabetic subjects, and the diagnostic limitations of traditional questionnaires in this patient population, the availability of specific screening tools of proven validity is fundamental in ensuring an early identification of the disorder and timely administration of treatment.

Overall, 21.8% of the sample studied, 33% of females and 11.1% of males, met the criteria for at least one diagnosis of ED according to DSM-5, including in this percentage the “Unspecified EDs and Other Specified EDs”. A lower percentage, 12.8% of the total sample, 17.5% of females and 8.3% of males, met the criteria for at least one diagnosis of ED according to DSM-IV, including “ED Not Otherwise Specified”. This finding of different rates of prevalence of EDs according to criteria adopted by DSM-IV or DSM-5, is in agreement with recent data from literature highlighting, in general, an increased ability of DSM-5 criteria to identify the presence of an ED, in addition to the acknowledged ability, linked to the higher degree of inclusivity of the diagnostic criteria, to formulate a “specified ED diagnosis”, thus reducing the rate of “non – specified ED diagnosis” [34].

The high prevalence of EDs identified at EDI-3 in the sample studied, is in agreement with data from literature reporting a high prevalence of EDs and DEB in diabetic patients, with rates exceeding those observed in the general population, and which vary according to the characteristics of the sample, to the rating tools applied and to the criteria used to formulate a diagnosis [1, 4, 6, 7, 13, 15]. ED prevalence rates according to DSM-5 are moreover higher compared to the rates of clinical risk of ED reported at EDI-3, thus highlighting the increased ability of a structured interview such as the amended version of module H of SCID-1, to identify cases of ED compared to those detected using a self-administered tool lacking specificity for the assessment of EDs in diabetes. In our sample a significantly higher prevalence of ED according to criteria adopted by DSM-IV and DSM-5, a significantly increased risk of ED at EDI-3, and higher scores at DEPS-R were found amongst females. Accordingly, there is a consensus in literature establishing that females, both adolescents and adults, have a higher prevalence and an increased risk of ED even if prevalence remains relatively high amongst males [42, 43]. A possible hypothesis advanced for this discrepancy may be attributed to the diagnostic criteria applied, which fail to consider the specific gender-related symptoms of ED, for example the expression of dissatisfaction with body image [44]. Moreover, the fact that the majority of ED studies have been conducted on exclusively, or prevalently, female samples, tends to complicate or even contradict the picture of clinical characteristics of males with ED, thus hampering identification of these disorders [45]. The higher scores obtained at DEPS-R by females are in agreement with the findings of previous studies conducted to validate the scale [29, 31, 32].

The Italian version of DEPS-R displayed an adequate degree of structural stability even in different linguistic and social settings (Table 3). The latent structure of subscales was found to fully conform to the latent dimensions referred for factors 1 2 and 3 by Wisting et al. [32]. Indeed, the three latent factors relating to dimensions “maladaptive eating habits” (factor 1), “preoccupation with thinness or weight” (factor 2), and “concept of maintaining high blood glucose values to lose weight” (factor 3) were all confirmed [32] (Table 2). Overall, the Italian version of the scale demonstrated a good degree of reliability, a good homogeneity and a good reproducibility, confirming the good psychometric properties of the original version, with the addiction of test-retest reliability assessment, which was not contemplated for the English version. Moreover, in this study, analysis of the DEPS-R scale in relation to EDI-3, a specific tool for the assessment of ED, and the use of a structured clinical interview, H module of SCID-1 and amended H module, allowed us to overcome some of the more significant limitations of previous validation studies.

Markowitz et al. [29] validated DEPS-R in a pediatric sample of boys and girls with type 1 diabetes, obtaining a good internal consistency and good construct validity by means of comparison with areas that may be affected by the presence of a DEB (positive correlation with age, BMI standardized for age, sex and hemoglobin A1C), in addition to a good external validity based on clinicians’ reports of insulin restriction in their patients. In the authors’ opinion, one of the main limitations of the study was the lack of comparison with a specific rating tool for ED, the small sample size (112 youths), and the age range of the sample (13–19 years), which were not sufficient to allow results to be generalized to other age groups [29]. Two years later, in extending validation of the scale to a larger sample of 770 young patients (11–19 years) with type 1 diabetes, Wisting et al. [32] revealed a good correlation of DEPS-R with the Eating Attitudes Test – (EAT-12), a general rating tool for EDs. A study conducted to validate the German version of the scale was carried out on 246 subjects with type 1 diabetes (11–19 years), showing a good internal consistency and a good construct validity for the scale, with a significant correlation between DEPS-R and two general screening tools for ED (SCOFF and the Eating Disorder Examination Questionnaire – EDE-Q) [31]. Similar to the findings of Wisting et al. and Saßmann et al. [31, 32], the DEPS-R scale also displayed a good concurrent validity in our study. Namely, a significant correlation was seen between total score at DEPS-R and the 3 ED risk scales of EDI-3 (“Body dissatisfaction”, “Drive for Thinness” and “Bulimia”) and between DEPS-R and score obtained at EDRC. Factors 1, 2 and 3 of DEPS-R were found to correlate with both ED risk scales and with EDRC score. Indeed, based on scores at EDI-3, subjects with a higher score at DEPS-R were characterized by more disturbed eating behaviors and increasing concerns about eating and body image. At the same time, subjects who met criteria for the diagnosis of at least one ED according to DSM-IV and/or DSM-5 achieved significantly higher mean scores at DEPS-R compared to subjects without this diagnosis. This finding highlights the ability of the scale to identify both subjects at risk of ED and subjects affected by full-blown ED.

Accordingly, incremental validity analysis for DEPS-R versus EDI-3 revealed the high validity of DEPS-R in predicting a diagnosis of ED in line with DSM-5 criteria, highlighting how use of this tool in combination with EDI-3 would produce a synergic effect in predicting diagnosis of ED, thus reducing the risk of detection of false negatives (Table 7). In support of the added value provided by this tool for the investigation of EDs in subjects affected by diabetes, the brevity of the scale, making it easy to use in a clinical setting, and the correlation of DEPS-R scores with metabolic imbalance in diabetic patients, should be underlined (Table 6). Higher score at DEPS-R was significantly related to higher HbA1C value and BMI median score in the sample as a whole and in males, females and subjects with type 1 diabetes, whereas the correlation between the EDRC score and the HbA1C was not significant. These results, in line with those reported by Saßmann et al., support the utility of the DEPS-R, a specific screening tool, to detect unhealthy practices of weight control leading to poor metabolic control in people with insulin treated diabetes [31].

Moreover, the higher representativeness of the sample in terms of age, following extension of the study to include subjects aged from 13 to 55 years, would appear to promote extension of validity of the questionnaire to larger samples of adolescent and adult patients affected by insulin-treated diabetes. Overall, the paucity of subjects with type 2 diabetes, respect to those affected by type 1 diabetes, does not allow the extension of the validation of the scale to this group. However, the significant more representation in this study of subjects affected by type 1 diabetes, respect to those affected by type 2 diabetes, reflects what is generally observed in clinical samples of insulin-treated subjects. In fact while the whole of patients affected by type 1 diabetes are treated with insulin, the treatment of type 2 diabetic patients include insulin only when strategies such as weight reduction, diet and oral medications fail to control diabetes. A further extension of the study to a larger group of patients affected by type 2 diabetes would be required to confirm extension of the validity of the scale to this diagnostic group.

The data should be interpreted in the light of several limitations, including the absence of an untreated sample of diabetic patients and the absence of a non-diabetic sample as control groups and the paucity of subjects with type 2 diabetes that does not allow the extension of the validation of the scale to this group.

## Conclusions

In conclusion, the present study conducted on a representative sample of insulin-treated adolescent and adult patients with both type 1 and type 2 diabetes, confirms the reliability and validity of the Italian version of the DEPS-R scale, showing a good construct validity, a good internal consistency, and a good degree of reproducibility. Analysis of the scale in relation to EDI-3, a specific tool used to assess EDs, and accompanied by use of a structured clinical interview in the diagnosis of ED, has highlighted the ability of the scale to identify both subjects at risk of ED and those affected by full-blown ED. In view of the significance and implications of EDs in patients affected by diabetes, with the associated health risks and singular presentation of EDs that render the results obtained by traditional ED screening tools unreliable, the availability of specific screening tools of proven validity is crucial to ensure a correct and rapid clinical-diagnostic classification and timely treatment of this co-morbidity.

## Additional file

Additional file 1: Diabetes eating problems survey – revised. (DOCX 14 kb)
