# Supplementary material for: Assessment of eating disorders with the diabetes eating problems survey – revised (DEPS-R) in a representative sample of insulin-treated diabetic patients: a validation study in Italy
Source: BMC Psychiatry. 2017 Jul 19;17:262. doi: 10.1186/s12888-017-1434-8 (PMC5518128; doi:10.1186/s12888-017-1434-8)
Supplement: Additional file 1: — Diabetes eating problems survey – revised. (DOCX 14 kb) [file 12888_2017_1434_MOESM1_ESM.docx]

**Additional file 1**

**DIABETES EATING PROBLEMS SURVEY – REVISED**

Instructions: The following statements concern attitudes, feelings, and behaviors relating to your eating habits and your diabetes care. For each statement, decide which answer best suits you by choosing among NEVER, RARELY, SOMETIMES, OFTEN, USUALLY and ALWAYS and mark your choice with a cross within the corresponding box. There are no right or wrong answers; give the answer that seems to describe you best at present.

| Never | Rarely | Sometimes | Often | Usually | Always |
| --- | --- | --- | --- | --- | --- |
| 0 | 1 | 2 | 3 | 4 | 5 |

____ 1) Losing weight is an important goal to me.

____ 2) I skip meals and/or snacks.

____ 3) Other people have told me that my eating is out of control.

____ 4) When I overeat, I don’t take enough insulin to cover the food.

____ 5) I eat more when I am alone than when I am with others.

____ 6) I feel that it’s difﬁcult to lose weight and control my diabetes at the same time.

____ 7) I avoid checking my blood sugar when I feel like it is out of range.

____ 8) I make myself vomit.

____ 9) I try to keep my blood sugar high so that I will lose weight.

____ 10) I try to eat to the point of spilling ketones in my urine.

____ 11) I feel fat when I take all of my insulin.

____ 12) Other people tell me to take better care of my diabetes.

____ 13) After I overeat, I skip my next insulin dose.

____ 14) I feel that my eating is out of control .

____ 15) I alternate between eating very little and eating huge amounts.

____ 16) I would rather be thin than to have good control of my diabetes.
